# Pilot Study on Alteration of LA-MRSA Status of Pigs during Fattening Period on Straw Bedding by Two Types of Cleaning

**DOI:** 10.3390/antibiotics10050521

**Published:** 2021-05-02

**Authors:** Hannah Schollenbruch, Iris Kobusch, Iris Schröter, Alexander Mellmann, Robin Köck, Marc Boelhauve

**Affiliations:** 1 Department of Agriculture, South Westphalia University of Applied Sciences, 59494 Soest, Germany; schollenbruch.hannah@fh-swf.de (H.S.); kobusch.iris@fh-swf.de (I.K.); schroeter.iris@fh-swf.de (I.S.); 2 Institute of Hygiene, University of Münster, 48149 Münster, Germany; Alexander.Mellmann@ukmuenster.de (A.M.); kockr@uni-muenster.de (R.K.); 3 Institute of Hygiene, DRK Kliniken Berlin, 14050 Berlin, Germany

**Keywords:** MRSA, MRSA-status alteration, decolonization

## Abstract

In countries with professional pig husbandry in stables, the prevalence of livestock-associated (LA) methicillin-resistant *Staphylococcus aureus* (MRSA) on farms has remained high or has further increased in recent years. Simple measures to reduce LA-MRSA among pigs have not yet been successfully implemented. The aim of this pilot study is twofold: first, to examine how the LA-MRSA status of LA-MRSA positive fattening pigs at the date of housing changes over the fatting period on straw bedding and, second, whether this change could be influenced by the quality of cleaning and disinfection (C&D). For this purpose, 122 animals are individually tested for LA-MRSA carriage at five sequential time points comparing pigs housed on a farm using straw bedding plus C&D (*n* = 59) vs. straw bedding plus simple cleaning (*n* = 63). At the time of housing, all animals in both groups are LA-MRSA positive. This status changes to 0% in the group with simple cleaning until the end of fattening and 28% in the C&D group. LA-MRSA in environmental and air samples is also reduced over the fattening period. The results indicate that keeping pigs on straw might be one way to reduce LA-MRSA during the fattening period with simple cleaning appearing to be more beneficial than C&D. Further investigations are necessary to determine the influencing factors more precisely.

## 1. Introduction

Methicillin-resistant *Staphylococcus aureus* (MRSA) has been detected in livestock (LA-MRSA), particularly in pigs [1]. The very frequent use of antibiotics in animal husbandry has been discussed as a plausible cause of continuous selection pressure facilitating the spread of LA-MRSA in animals and humans [2]. Pig farmers can be directly affected by LA-MRSA from their own livestock. They are frequently (up to 80% of pig farmers) colonized by LA-MRSA in the nares and might even become infected [3], as reviewed in [4]. Since 2006, the influence of LA-MRSA colonization caused by contact with livestock on the epidemiology of MRSA in hospitals located in rural areas and on human infections has been clearly demonstrated [5,6]. A long-term decolonization of persons with continuous livestock contact has so far been unsuccessful, as these persons have been repeatedly recolonized, if LA-MRSA has still been detectable in the stable [4,7]. Besides the impact on directly exposed persons, the risk of LA-MRSA contamination in meat for the community has been extensively discussed [8,9], as overviewed in [10].

As a result, control points were sought to limit the transmission of resistant pathogens “from stable to table” and interventions to reduce the colonization of livestock were demanded. To date, only the intensive culling of LA-MRSA-positive livestock in Norway has proven to be successful [11], and only for a short time. This opportunity is neither possible in Germany (transit country in Europe) nor has the short-term success in Norway been socially accepted [12]. Due to frequent transport of pigs [13] and incompletely implemented barrier measures (e.g., separation of LA-MRSA-colonized farmers from LA-MRSA-negative animals) in Germany [10], consistent establishment of LA-MRSA-negative herds is difficult. Other interventions, such as the active decolonization of pigs using disinfectants or bacteriophages have been either unsuccessful or very laborious [14,15].

One aspect of the problem is that LA-MRSA is not only detected in the nares of animals, but also on surfaces, such as stable walls and equipment, as well as in the air and in dust samples [16,17,18]. Although well-conducted cleaning and disinfection can be effective in decontaminating the farm environment, new animals that are brought to this setting will recontaminate the environment, including the air, within a few minutes, if they carry LA-MRSA [16].

Previous literature suggests that there are differences in the occurrence of LA-MRSA between conventional pig farms and alternative farms (e.g., reduced number of pigs, straw bedding) with alternative farms being largely free of LA-MRSA [19,20], while on conventional farms, LA-MRSA antibiotic resistance is found to a large extent [17,21]. However, the reasons for these differences have not been clearly investigated. Of course, factors like antibiotic treatment, production type, herd size and intensity of animal husbandry may play an important role [22]. Another major difference between conventional and alternative farms (organic farms or farms participating in specific animal welfare programs) is that the latter often use straw as a bedding material in the stables, while conventional farms often use slatted floors. This aspect might also have an impact on LA-MRSA: while slatted floors are apparently easier to clean, one can hypothesize that straw is an organic material containing a variety of bacteria [23] that might reduce LA-MRSA in the environment by competitive effects.

In this observational pilot study, we assess two groups of pigs that carry LA-MRSA when admitted to a fattening farm to understand how LA-MRSA carriage is affected over time. Using bedding on straw, this farm differs from conventional husbandry and can be classified as alternative farming. In addition, we observe whether the technique used to clean the stables before housing the pigs (simple cleaning only vs. cleaning and disinfection) has an impact on nasal LA-MRSA-carriage, as well as the surfaces and air in the stable. The investigations take place over an entire fattening period.

## 2. Results

### 2.1. LA-MRSA Carriage among Pigs

**Figure 1:**
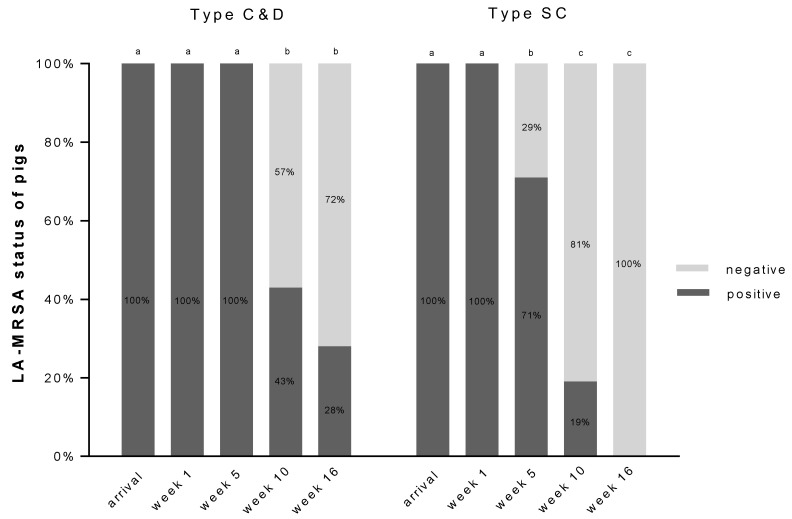
Illustration of the percentage of LA-MRSA detection in the pigs in type C&D (*n* = 63 animals, cleaning and disinfection; *n* = 309 samples) and in type SC (*n* = 59 animals, simple cleaning; *n* = 292 samples) at five time points during fattening; Cochran’s Q Test, significant differences are indicated by letters (a–c) (*p* < 0.05).

Screening of the fattening pigs during housing-in showed nasal LA-MRSA carriage of all animals (*n* = 122). Genotyping (*n* = 61, *n* = 32 type C&D, *n* = 29 type SC) demonstrated that all LA-MRSA isolates exhibited spa types indicative of an LA-MRSA CC398 clonal lineage, i.e., spa types t011 (31%) and t034 (69%); both spa types were present in both groups of animals. During the fattening period, the pigs and the environment were analyzed periodically. Individual screening of type C&D showed that all pigs carried LA-MRSA when they arrived, without changes in weeks one and five. Later, beginning in week 10, the pigs showed several negative LA-MRSA reports (Figure 1). At the end of the fattening phase, 72% of the pigs in type C&D were LA-MRSA negative. Animals in type SC also showed a change in LA-MRSA status. However, the proportion of LA-MRSA negative animals was already higher after five weeks compared to the pigs in type C&D (Figure 1). By the end of the fattening period, all animals in type SC were LA-MRSA negative.

The differences between both groups were significant from weeks five (*p* < 0.01), 10 (*p* = 0.009) and 16 (*p* < 0.01) (chi-squared test).

Animal-specific analysis of LA-MRSA carriage status showed that some animals (*n* = 7, only in type C&D) remained constantly LA-MRSA-positive, while others were either intermittently (*n* = 15, in both types) or constantly LA-MRSA-negative after five weeks (*n* = 12, only in type SC) (Table 1).

### 2.2. LA-MRSA in the Environment

**Figure 2:**
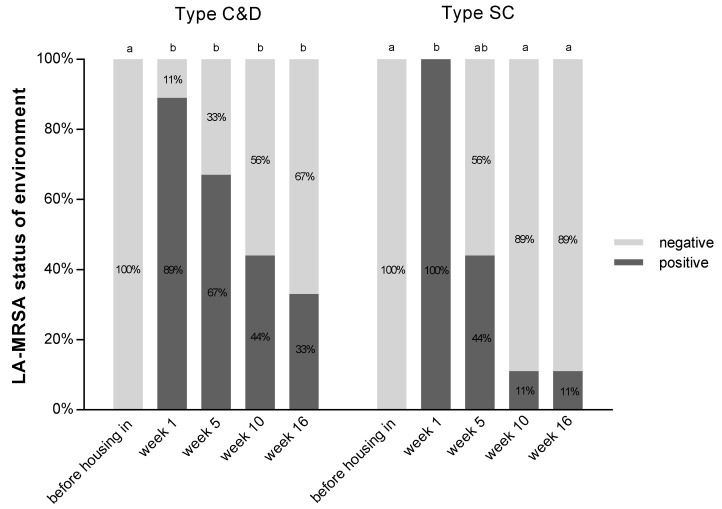
Percentage of LA-MRSA positive and negative environmental samples (surfaces, without straw samples) in type C&D (*n* = 55) and type SC (*n* = 55) during fattening period; Cochran’s Q Test, significant differences are indicated by letters (a, b) (*p* < 0.01).

Analysis of the environmental samples, including straw samples, showed that before the pigs were housed, LA-MRSA was not detectable in either barn. After one week, more than 90% of the environmental samples in type C&D were already LA-MRSA positive, while in type SC, all samples were positive. From week five onward, a reduction in the detection rates of LA-MRSA was noticed. Half of the samples in type SC were LA-MRSA negative after five weeks (type C&D, 33%) and by the end of the fattening period, 89% of all samples were MRSA negative. In type C&D, 33% of the environmental samples were still LA-MRSA positive after 16 weeks (Figure 2). Analysis of the environmental samples showed no differences between the two types.

Analyses of the surfaces in the two groups showed that in the phase of decreasing LA-MRSA detection (weeks 5 to 16 in both groups), some surfaces remained constant after reaching LA-MRSA-negative status, while others showed intermittent LA-MRSA findings (Table 2).

### 2.3. LA-MRSA Status of Air Samples

**Figure 3:**
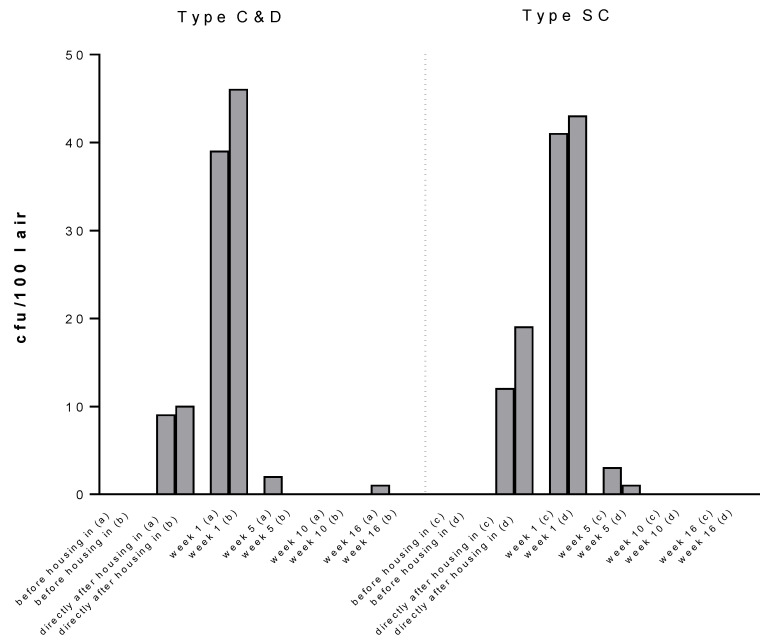
Collected air samples during fattening phase. Samples were collected at two different points inside the stables (front and back) at described time points. a and c (front and back samples, respectively, type C&D); b and d (front and back samples, respectively, type SC). The corresponding sample locations are shown comparatively.

Air samples were taken in both stables during the fattening phase and there were no LA-MRSA colonies on selective media before housing-in. Already after 15 min animal contact, the stable air contained over 9 or 10 cfu/100 L in type C&D and 12 or 19 cfu/100 L in type SC. These values reached a peak in both types in week one and decreased constantly in the following weeks (Figure 3).

## 3. Discussion

The fattening pigs assigned to the two types of treatment, i.e., different types of cleaning and disinfection before housing, had the same initial situation. Both groups of animals were born on the same farm and were tested directly upon arrival. They were all LA-MRSA-positive and carried typical LA-MRSA spa types associated with the clonal lineage CC398. All animals were then placed in a barn with an initially LA-MRSA negative environment. During the fattening period, a clear trend towards a reduction in the occurrence of LA-MRSA was observed in both groups of pigs, with the reduction starting earlier in the SC group. While at week five, the entire animal group in type C&D was still positive for the detection of LA-MRSA, 29% of the animals in type SC were already LA-MRSA negative. From week 10, a continued reduction was observed in both groups.

The considerable differences in LA-MRSA prevalence at 16 weeks between the two types may be due to thorough decontamination of bacteria from the previous fattening phase (type C&D). A well-performed C&D will reduce the bacterial microbial load by a factor of 10,000 [24,25]. This influence of the initial bacterial concentration at the time of stabling can be considered substantial and represents the only difference between the two groups of animals considered. Both groups did not differ in origin, feeding, ventilation or other housing conditions. The presumed higher bacterial load at the beginning of the fattening period in type SC (week five) may have caused the animals or the uncontaminated straw to be more likely to compete against LA-MRSA.

Hence, the main finding of this study is that it is possible that pigs may be decolonized from LA-MRSA after being placed in an LA-MRSA negative environment. Both barns where this was successfully done had three major differences compared with other conventional pig farms (with full slatted floors) in Germany: (1) there was open ventilation in both barns (otherwise, mostly forced ventilation), (2) lower animal stocking density (1.1 m^2^ per animal, instead of 0.75 m^2^ [26]) and (3) straw bedding on a flat floor (instead of a full slatted floor). Unfortunately, a comparison of these two investigated groups with housing on a slatted floor is not possible due to the differences in housing types mentioned. In addition, the requirements for this comparison should be addressed to, e.g., identical genetics, rearing operation and feeding technique. The possibility of LA-MRSA status change and differences between farms are known, but influencing factors are unclear [27,28,29,30]. Studies of [27] indicate a similar trend in colonization over the fattening period of four Swiss farms. Swiss husbandry clearly differs from other EU husbandries, because of higher standards for animal husbandry, such as lower stocking density or no lying areas on slatted floor [31]. Possible factors influencing this change of state with regard to LA-MRSA detection, like herd size and antibiotic treatment, have already been suspected in previous studies [21,32,33,34,35], but subsequent tracing of individual animals has not been carried out. Studies with individual animal tracking cannot highlight clear influencing factors, except for antimicrobial use [27,29,30]. [29] suggests a link between colonization with methicillin-sensitive *Staphylococcus aureus* (MSSA) and LA-MRSA (early colonization of piglets with MSSA could prevent colonization with LA-MRSA), but [28] cannot confirm this.

Most of the studies [21,32,33,34,35] highlighted risk factors leading to LA-MRSA colonization and focused less on the process of decolonization of animals. The study of [32] covered the floor condition in pig farming, but did not describe this influencing factor in more detail. Other studies excluded alternative husbandry systems from their investigations [35]. Therefore, the main difference to existing studies is the lack of individual animal tracking in combination with factors reducing LA-MRSA during a lifetime period.

It was shown in this study that on the one hand, colonization of pigs with LA-MRSA is a highly dynamic process, which on the other hand, depends on environmental factors. Whether these influencing factors can only be narrowed down to differential preparation of the barn (intensive C&D vs. simple cleaning without disinfection) should be determined in more detail in further investigations. The decrease in LA-MRSA detection in both groups suggests that straw bedding and the close contact of animals with bedding material due to species-specific behavior might play a decisive role in this process. Indications of this can be found in previous studies [19,20].

The effects of competitive bacteria have been studied since 1973 [36]. Competition leads to reduces availability of nutrients, resulting in a lower growth rate of the bacterial species that are aimed to be minimized. The grade of competition depends on the concentration of the competitive bacteria [37]. *Lactobacillus* sp. is one of many commensal epiphytes on plants like straw [38]. Furthermore, bacteria used as competitors, like lactobacilli, cause a lowering of the pH, which also contributes to inhibiting the growth of other bacteria [39]. Exemplarily, 84 out of 104 *Lactobacillus* strains showed an effect on the growth of *Clostridium perfringens* [40]. Other direct effects are possible, as well [39]. The competitive effect on MRSA growth has already been described on the basis of laboratory analyses [41]. However, this has not yet been described with regard to LA-MRSA and also not in pig farming, in particular, with the effect of complete displacement of LA-MRSA from colonized animals.

It can be assumed that species-specific active interaction with the straw bedding and the epiphytic microorganisms contained therein may have exerted an influence on the microbiome of the animals. In this context, it is conceivable that there could be a competitive situation between the epiphytic and animal-specific microbiome, and LA-MRSA could no longer retain a place in the nasopharyngeal tract of the pigs during this competitive struggle.

These microbiome investigations were not part of this study and are currently being performed to describe approaches to move swine husbandry to an LA-MRSA-free state. These approaches do not necessarily imply a complete switch to straw bedding; it is also conceivable that efforts to achieve LA-MRSA absence could be limited to sow management, since the available cleaning and disinfection measures can be used to represent a subsequent colonization of the animals in rearing or fattening operations. In this context, the MRSA status of the persons working in these farms should be taken into account to avoid a secondary colonization of the animals.

In conclusion, this pilot study shows that straw bedding for pigs and modified routines in C&D could be a possible starting point for further studies to reduce or eliminate LA-MRSA colonization in pigs.

## 4. Material and Methods

### 4.1. Organization of the Farm/Stable

This study was conducted on a conventional pig fattening farm in Germany. The pig farm had two fattening barns with straw bedding. The barns were equipped with seven pens each, housing an average of 60–63 animals. Both pens had front-mounted bowl drinkers and a pulp feeder. Supply air was provided through large windows, each extending above the control aisle on one side of the complete compartment. Both barns had a concrete floor without gaps and compartment walls partly made of concrete and wood. Bedding consisted of a straw bale placed in the center of the pen, which was actively distributed by the pigs. New straw bales were added after about 8–10 days.

Normally, after the previous animal group had moved out, the barn was empty for approximately 7–10 days. Usually, the farmer only cleaned the stables and did not perform disinfection. For this study, the two barns were cleaned or also disinfected, differently: In type 1, thorough cleaning of the pens was performed followed by disinfection (C&D). In type 2, only simple cleaning with water was carried out and no additional disinfection was performed (simple cleaning = SC). In detail, in type C&D, manual removal of partially dried manure and straw bedding, soaking of the residual dirt with water over several hours with a high-pressure cleaner, pre-cleaning of the compartment with a high-pressure cleaner, followed by a foam cleaning phase with sodium hydroxide (product: Menno Clean, Menno Chemie, Norderstedt, Germany) and an intensive cleaning phase with a high-pressure cleaner were performed. After a drying time of approximately 18 h, all surfaces were treated with foam disinfectant (product: Venno Vet 1 Super, Menno Chemie, Norderstedt, Germany). This work was carried out by employees of the South Westphalia University of Applied Sciences who were experienced in the C&D procedure described above, as this procedure was not part of the routine operating procedure at this farm. 

In type SC, removal of straw bedding and manure also took place. Here, the cleaning was carried out by the farmer (according to the usual procedure), whereby a soaking of the dirt was also carried out. Here, the application of alkaline foam cleaner was skipped. In addition, the cleaning phase was conducted with the high-pressure cleaner in about half the time (compared to type C&D).

This farm purchased piglets with an estimated weight of approximately 25 kg from a livestock trader. Each group of animals studied (63 pigs in type C&D, 59 in type SC) came from the same source and was housed at the same time.

### 4.2. Sample Collection

**Figure 4:**
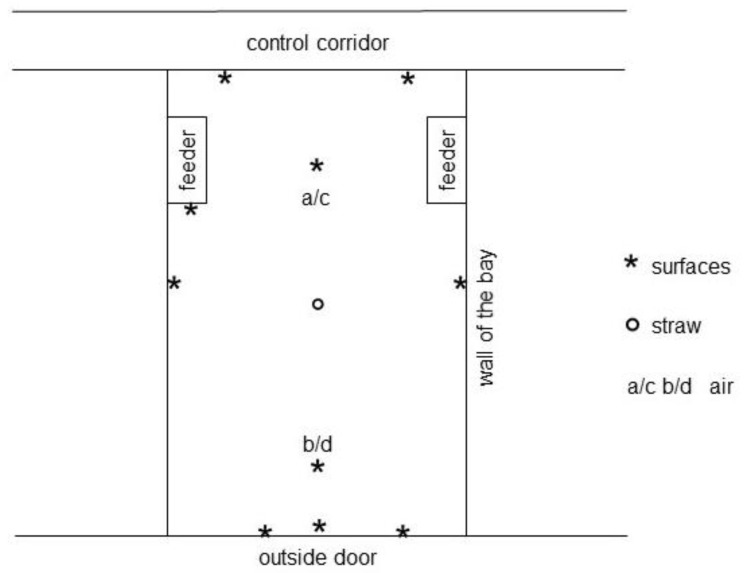
Schematic overview of the environmental sampling locations in the bay.

For traceable sample collection, all pigs were tagged individually with transponders (MS Quick Transponder FDX, MS Shippers, Bladel, The Netherlands). The nasal carriage of animals with LA-MRSA was documented directly after being transported to the new stable. As such, a swab (swab with amies liquid, VWR) was taken from the inner side of the nostrils during housing-in and after 1, 5, 10 and 16 weeks (in total *n* = 601 samples). Moreover, the LA-MRSA contamination of the environment before housing-in and during the fattening period was investigated with swab samples at defined spots with animal contact and on dusted areas inside the stables; 20 environmental samples (20 cm^2^, 4 cm × 5 cm, including floor samples) were taken at each barn (Figure 4) before housing-in and nine environmental samples (without floor samples) were taken at each barn 1, 5, 10 and 16 weeks thereafter (*n* = 112). The number of environmental samples was identical in both groups investigated. Additionally, the surrounding air (*n* = 20) was also collected at each time point (MicroBio MB2, Cantium Scientific, Dartford, UK) by aspirating 100 L of air within one minute onto a chromID™ MRSA SMART Agar (bioMérieux, Nurtingen, Germany). The agar plate was then immediately closed in the barn until further incubation at the laboratory. Straw samples during the fattening period (*n* = 10) and from the stock sample (*n* = 2) were taken. Surface and air samples were collected at defined locations (Figure 1) all over the stables at the same time points as the animal samples. The sampling locations and methods of sampling are listed in detail in Table 3.

### 4.3. Phenotypic MRSA Detection and Genotyping

For microbiological analysis, the nasal and environmental swabs were transferred in 9 mL Mueller Hinton broth + 6.5% NaCl (Mediaproducts BV, Groningen, The Netherlands) and incubated at 37 °C for 18 ± 2 h for enrichment of staphylococci. From these samples, 500 µL were added to 5 mL Tryptone soya broth + cefoxitin/aztreonam (Mediaproducts BV, Groningen, The Netherlands) and grown over 18 ± 2 h at 37 °C for MRSA-enrichment. Subsequently, 10 µL of the enriched cultures were inoculated on chromID™ MRSA SMART Agar (bioMérieux, The Nurtingen, Germany) and incubated at 37 °C for 24 h. Straw samples were chopped and 5 g filled with buffered peptone water (Merck, Darmstadt, Germany) in a ratio of 1:10 was blended in a stomacher for 30 s at 230× *g*. Samples were then further processed like the nasal and environmental swabs (see above).

Plates of air samples were directly incubated as described above. Typical colorimetric colonies grown on chromID™ MRSA SMART Agar (bioMérieux, Nurtingen, Germany) were selected. Spa typing was performed following a previously described protocol [42]. Briefly, we amplified an internal fragment of the spa gene and sequenced it subsequently. For further analysis, the repeat region of *spa* was extracted and the spa type determined using the Ridom StaphType software version 2.0 (Ridom GmhbH, Münster, Germany).

### 4.4. Statistical Analysis

For analysis of non-parametric variables, calculated mean values were tested for statistical significance between sampling time points by Cochran’s Q Test. The data were analyzed descriptively using IBM SPSS Statistics 21 and Excel 2016. For the illustration of the figures, GraphPad Software (San Diego, CA, USA) was used.

## 5. Conclusions

This study focused on the dynamics of LA-MRSA colonization in pigs kept on straw during the fattening phase. The influence of two different cleaning intensities—(i) a very thorough cleaning and disinfection (C&D) and (ii) a simple cleaning without disinfection (SC = simple cleaning)—were determined. The animals investigated came from the same farm and were housed in experimental barns at the same time. These barns were structurally separated. The animals, the environment and the straw were examined directly at stabling and at four further time points. Individual animal evaluations showed that the animals in group SC were free of LA-MRSA after 16 weeks, while group C&D was still 28% colonized. Both groups of animals were 100% LA-MRSA positive at the time of housing, so both cleaning procedures led to a significant decrease in colonization.

The environmental samples also showed a comparable decrease in LA-MRSA colonization. It can be assumed that the freedom from LA-MRSA in group SC is linked to the initial bacterial load. The simple cleaning might have been associated with a transfer of substantially more bacteria from the preceding pig fattening group and thus may have promoted the change in LA-MRSA status due to the competitive effects of other bacteria on MRSA. It is likely that the behavior of the pigs in the straw (e.g., burrowing) resulted in mixing the “old” bacteria with the epiphytic bacteria of the straw, allowing the change in LA-MRSA status to begin earlier. Nevertheless, the R&D group also shows a similar decrease in LA-MRSA colonization, starting with a time delay compared to the C&D group.

In conclusion, this pilot study shows that straw bedding for pigs, in combination with different types of cleaning and disinfection, could be a possible starting point for further studies aiming to investigate opportunities to reduce or eliminate LA-MRSA colonization in pigs.

## Figures and Tables

**Table 1:** Individual animal tracking and LA-MRSA status from arrival to week 16 of fattening phase (pos = positive; neg = negative).

Animals in Type C&D						Animals in Type SC					
Animal ID	Arrival	Week				Animal ID	Arrival	Week			
		1	5	10	16			1	5	10	16
603	pos	pos	pos	pos	pos	328	pos	pos	pos	pos	neg
607	pos	pos	pos	pos	pos	346	pos	pos	pos	pos	neg
615	pos	pos	pos	pos	pos	347	pos	pos	pos	pos	neg
627	pos	pos	pos	pos	pos	381	pos	pos	pos	pos	neg
653	pos	pos	pos	pos	pos	387	pos	pos	pos	pos	neg
693	pos	pos	pos	pos	pos	397	pos	pos	pos	pos	neg
697	pos	pos	pos	pos	pos	303	pos	pos	neg	pos	neg
606	pos	pos	pos	neg	pos	309	pos	pos	neg	pos	neg
635	pos	pos	pos	neg	pos	335	pos	pos	neg	pos	neg
639	pos	pos	pos	neg	pos	352	pos	pos	neg	pos	neg
646	pos	pos	pos	neg	pos	368	pos	pos	neg	pos	neg
647	pos	pos	pos	neg	pos	302	pos	pos	pos	neg	neg
659	pos	pos	pos	neg	pos	304	pos	pos	pos	neg	neg
677	pos	pos	pos	neg	pos	306	pos	pos	pos	neg	neg
682	pos	pos	pos	neg	pos	307	pos	pos	pos	neg	neg
683	pos	pos	pos	neg	pos	310	pos	pos	pos	neg	neg
691	pos	pos	pos	neg	pos	319	pos	pos	pos	neg	neg
628	pos	pos	pos			322	pos	pos	pos	neg	neg
644	pos	pos				324	pos	pos	pos	neg	neg
604	pos	pos	pos	pos	neg	327	pos	pos	pos	neg	neg
612	pos	pos	pos	pos	neg	330	pos	pos	pos	neg	neg
616	pos	pos	pos	pos	neg	333	pos	pos	pos	neg	neg
620	pos	pos	pos	pos	neg	336	pos	pos	pos	neg	neg
621	pos	pos	pos	pos	neg	340	pos	pos	pos	neg	neg
622	pos	pos	pos	pos	neg	343	pos	pos	pos	neg	neg
625	pos	pos	pos	pos	neg	345	pos	pos	pos	neg	neg
642	pos	pos	pos	pos	neg	348	pos	pos	pos	neg	neg
645	pos	pos	pos	pos	neg	349	pos	pos	pos	neg	neg
655	pos	pos	pos	pos	neg	353	pos	pos	pos	neg	neg
657	pos	pos	pos	pos	neg	355	pos	pos	pos	neg	neg
663	pos	pos	pos	pos	neg	359	pos	pos	pos	neg	neg
664	pos	pos	pos	pos	neg	360	pos	pos	pos	neg	neg
665	pos	pos	pos	pos	neg	361	pos	pos	pos	neg	neg
674	pos	pos	pos	pos	neg	362	pos	pos	pos	neg	neg
679	pos	pos	pos	pos	neg	369	pos	pos	pos	neg	neg
680	pos	pos	pos	pos	neg	371	pos	pos	pos	neg	neg
696	pos		pos	pos	neg	375	pos	pos	pos	neg	neg
698	pos	pos	pos	pos	neg	380	pos	pos	pos	neg	neg
602	pos	pos	pos	neg	neg	382	pos	pos	pos	neg	neg
605	pos	pos	pos	neg	neg	384	pos	pos	pos	neg	neg
613	pos	pos	pos	neg	neg	385	pos	pos	pos	neg	neg
614	pos	pos	pos	neg	neg	395	pos	pos	pos	neg	neg
618	pos	pos	pos	neg	neg	396	pos	pos	pos	neg	neg
619	pos	pos	pos	neg	neg	400	pos	pos	pos	neg	neg
638	pos	pos	pos	neg	neg	331		pos	pos	neg	neg
640	pos	pos	pos	neg	neg	388	pos	pos	pos	neg	
643	pos	pos	pos	neg	neg	393	pos	pos	pos	neg	
648	pos	pos	pos	neg	neg	311	pos	pos	neg	neg	neg
649	pos	pos	pos	neg	neg	314	pos	pos	neg	neg	neg
651	pos	pos	pos	neg	neg	318	pos	pos	neg	neg	neg
658	pos	pos	pos	neg	neg	344	pos	pos	neg	neg	neg
672	pos	pos	pos	neg	neg	351	pos	pos	neg	neg	neg
673	pos	pos	pos	neg	neg	366	pos	pos	neg	neg	neg
675	pos	pos	pos	neg	neg	367	pos	pos	neg	neg	neg
676	pos	pos	pos	neg	neg	376	pos	pos	neg	neg	neg
678	pos	pos	pos	neg	neg	377	pos	pos	neg	neg	neg
681	pos	pos	pos	neg	neg	378	pos	pos	neg	neg	neg
685	pos	pos	pos	neg	neg	390	pos	pos	neg	neg	neg
687	pos	pos	pos	neg	neg	399	pos	pos	neg	neg	neg
689	pos	pos	pos	neg	neg						
690	pos	pos	pos	neg	neg						
694	pos	pos	pos	neg	neg						
699	pos	pos	pos	neg	neg						

**Table 2:** Individual environmental samples and LA-MRSA during fattening period (pos = positive; neg = negative).

Environmental Samples in Type C&D						Environmental Samples in Type SC					
Sampling Location	Before Housing in	Week				Sampling Location	Before Housing in	Week			
		1	5	10	16			1	5	10	16
wall of the bay (control corridor, right)	neg	pos	neg	pos	pos	wall of the bay (control corridor, center)	neg	pos	neg	neg	neg
wall of the bay (control corridor, left)	neg	pos	pos	neg	pos	wall of the bay (right)	neg	pos	neg	neg	neg
wall of the bay (right)	neg	pos	pos	pos	neg	wall of the bay (left)	neg	pos	neg	neg	neg
wall of the bay (left)	neg	pos	pos	neg	neg	wall of the bay (outside door)	neg	pos	pos	neg	neg
wall of the bay (outside door)	neg	pos	pos	neg	neg	floor of the bay (control corridor)	neg	pos	neg	neg	pos
floor of the bay (control corridor)	neg	neg	pos	neg	neg	floor of the bay (center)	neg	pos	pos	neg	neg
floor of the bay (center)	neg	pos	neg	neg	neg	pipe above the wall of the bay (right)	neg	pos	pos	neg	neg
pipe above the wall of the bay (centered)	neg	pos	neg	pos	neg	pipe above the wall of the bay (left)	neg	pos	pos	neg	neg
wall of the feeder	neg	pos	pos	pos	pos	wall of the feeder	neg	pos	neg	pos	neg
straw (center)	neg	pos	pos	neg	neg	straw (center)	neg	pos	pos	neg	neg
air a (front)	neg	pos	pos	neg	pos	air c (front)	neg	pos	pos	neg	neg
air b (back)	neg	pos	neg	neg	neg	air d (back)	neg	pos	pos	neg	neg

**Table 3:** Overview of the environmental samples taken in type 1 and type 2.

Environmental Samples in the Bay of Type C&D	Environmental Samples in the Bay of Type SC
wall of the bay (control corridor, right)	wall of the bay (control corridor, center)
wall of the bay (control corridor, left)	wall of the bay (right)
wall of the bay (right)	wall of the bay (left)
wall of the bay (left)	wall of the bay (outside door)
wall of the bay (outside door)	floor of the bay (control corridor)
floor of the bay (control corridor)	floor of the bay (center)
floor of the bay (center)	pipe above the wall of the bay (right)
pipe above the wall of the bay (centered)	pipe above the wall of the bay (left)
wall of the feeder	wall of the feeder
straw (center)	straw (center)
air (front)	air (front)
air (back)	air (back)

## Data Availability

The data presented in this study are available on request from the corresponding author.

## References

[1] Beneke B., Klees S., Stührenberg B., Fetsch A., Kraushaar B., Tenhagen B.-A. (2011). Prevalence of methicillin-resistant Staphylococcus aureus in a fresh meat pork production chain. J. Food Prot..

[2] Hunter P.A., Dawson S., French G.L., Goossens H., Hawkey P.M., Kuijper E.J., Nathwani D., Taylor D.J., Teale C.J., Warren R.E. (2010). Antimicrobial-resistant pathogens in animals and man: Prescribing, practices and policies. J. Antimicrob. Chemother..

[3] Cuny C., Nathaus R., Layer F., Strommenger B., Altmann D., Witte W. (2009). Nasal colonization of humans with methicillin-resistant Staphylococcus aureus (MRSA) CC398 with and without exposure to pigs. PLoS ONE.

[4] Goerge T., Lorenz M.B., van Alen S., Hübner N.-O., Becker K., Köck R. (2017). MRSA colonization and infection among persons with occupational livestock exposure in Europe: Prevalence, preventive options and evidence. Vet. Microbiol..

[5] Köck R., Harlizius J., Bressan N., Laerberg R., Wieler L.H., Witte W., Deurenberg R.H., Voss A., Becker K., Friedrich A.W. (2009). Prevalence and molecular characteristics of methicillin-resistant Staphylococcus aureus (MRSA) among pigs on German farms and import of livestock-related MRSA into hospitals. Eur. J. Clin. Microbiol. Infect. Dis..

[6] Cuny C., Layer F., Werner G., Harmsen D., Daniels-Haardt I., Jurke A., Mellmann A., Witte W., Köck R. (2015). State-wide surveillance of antibiotic resistance patterns and spa types of methicillin-resistant Staphylococcus aureus from blood cultures in North Rhine-Westphalia, 2011–2013. Clin. Microbiol. Infect..

[7] Lozano C., Aspiroz C., Lasarte J.J., Gómez-Sanz E., Zarazaga M., Torres C. (2011). Dynamic of nasal colonization by methicillin-resistant Staphylococcus aureus ST398 and ST1 after mupirocin treatment in a family in close contact with pigs. Comp. Immunol. Microbiol. Infect. Dis..

[8] Bisdorff B., Scholhölter J.L., Claußen K., Pulz M., Nowak D., Radon K. (2012). MRSA-ST398 in livestock farmers and neighbouring residents in a rural area in Germany. Epidemiol. Infect..

[9] Alban L., Ellis-Iversen J., Andreasen M., Dahl J., Sönksen U.W. (2017). Assessment of the Risk to Public Health due to Use of Antimicrobials in Pigs—An Example of Pleuromutilins in Denmark. Front. Vet. Sci..

[10] Lassok B., Tenhagen B.-A. (2013). From pig to pork: Methicillin-resistant Staphylococcus aureus in the pork production chain. J. Food Prot..

[11] Elstrøm P., Grøntvedt C.A., Gabrielsen C., Stegger M., Angen Ø., Åmdal S., Enger H., Urdahl A.M., Jore S., Steinbakk M. (2019). Livestock-Associated MRSA CC1 in Norway; Introduction to Pig Farms, Zoonotic Transmission, and Eradication. Front. Microbiol..

[12] van Herten J., Bovenkerk B., Verweij M. (2019). One Health as a moral dilemma: Towards a socially responsible zoonotic disease control. Zoonoses Public Health.

[13] Gerritzen M.A., Hindle V.A., Steinkamp K., Reimert H.G.M., van der Werf J.T.N., Marahrens M. (2013). The effect of reduced loading density on pig welfare during long distance transport. Animal.

[14] Honegger J., Lehnherr H., Bachofen C., Stephan R., Sidler X. (2020). Feldversuch zur Eradikation von Methicillin-resistenten Staphylococcus aureus (MRSA) mittels Bakteriophagen in einem Schweinezuchtbetrieb. Schweiz. Arch. Tierheilkd..

[15] Pletinckx L., Dewulf J., De Bleecker Y., Rasschaert G., Goddeeris B., De Man I. (2013). Effect of a disinfection strategy on the methicillin-resistant Staphylococcus aureus CC398 prevalence of sows, their piglets and the barn environment. J. Appl. Microbiol..

[16] Kobusch I., Müller H., Mellmann A., Köck R., Boelhauve M. (2020). Single Blinded Study on the Feasibility of Decontaminating LA-MRSA in Pig Compartments under Routine Conditions. Antibiotics.

[17] Friese A., Schulz J., Hoehle L., Fetsch A., Tenhagen B.-A., Hartung J., Roesler U. (2012). Occurrence of MRSA in air and housing environment of pig barns. Vet. Microbiol..

[18] Schmithausen R.M., Kellner S.R., Schulze-Geisthoevel S.V., Hack S., Engelhart S., Bodenstein I., Al-Sabti N., Reif M., Fimmers R., Körber-Irrgang B. (2015). Eradication of methicillin-resistant Staphylococcus aureus and of Enterobacteriaceae expressing extended-spectrum beta-lactamases on a model pig farm. Appl. Environ. Microbiol..

[19] Cuny C., Friedrich A.W., Witte W. (2012). Absence of Livestock-Associated Methicillin-Resistant Staphylococcus aureus Clonal Complex CC398 as a Nasal Colonizer of Pigs Raised in an Alternative System. Appl. Environ. Microbiol..

[20] van de Vijver L.P.L., Tulinski P., Bondt N., Mevius D., Verwer C. (2014). Prevalence and molecular characteristics of methicillin-resistant Staphylococcus aureus (MRSA) in organic pig herds in The Netherlands. Zoonoses Public Health.

[21] Crombé F., Willems G., Dispas M., Hallin M., Denis O., Suetens C., Gordts B., Struelens M., Butaye P. (2012). Prevalence and antimicrobial susceptibility of methicillin-resistant Staphylococcus aureus among pigs in Belgium. Microbiol. Drug Resist..

[22] Pirolo M., Visaggio D., Gioffrè A., Artuso I., Gherardi M., Pavia G., Samele P., Ciambrone L., Di Natale R., Spatari G. (2019). Unidirectional animal-to-human transmission of methicillin-resistant Staphylococcus aureus ST398 in pig farming; evidence from a surveillance study in southern Italy. Antimicrob. Resist. Infect. Control.

[23] Wagner K.M., Schulz J., Kemper N. (2018). Examination of the hygienic status of selected organic enrichment materials used in pig farming with special emphasis on pathogenic bacteria. Porcine Health Manag..

[24] Hancox L.R., Le Bon M., Dodd C.E.R., Mellits K.H. (2013). Inclusion of detergent in a cleaning regime and effect on microbial load in livestock housing. Vet. Rec..

[25] Misra S., van Middelaar C.E., Jordan K., Upton J., Quinn A.J., De Boer I.J.M., O’Driscoll K. (2020). Effect of different cleaning procedures on water use and bacterial levels in weaner pig pens. PLoS ONE.

[26] Bundesgesetzblatt. https://www.bgbl.de/xaver/bgbl/start.xav?startbk=Bundesanzeiger_BGBl&jumpTo=bgbl106s2043.pdf#__bgbl__%2F%2F*%5B%40attr_id%3D%27bgbl106s2043.pdf%27%5D__1615288398869.

[27] Bangerter P.D., Sidler X., Perreten V., Overesch G. (2016). Longitudinal study on the colonisation and transmission of methicillin-resistant Staphylococcus aureus in pig farms. Vet. Microbiol..

[28] Fetsch A., Roesler U., Kraushaar B., Friese A. (2016). Co-colonization and clonal diversity of methicillin-sensitive and methicillin-resistant Staphylococcus aureus in sows. Vet. Microbiol..

[29] Nathaus R., Blaha T., Tegeler R., Meemken D. (2010). Intra-herdenprävalenz und Kolonisa-tionsdynamik von Methicillin-resistenten Staphylococcus aureus in zwei Schweine- zuchtbeständen. Berl. Munch. Tierarztl. Wochenschr..

[30] Weese J.S., Zwambag A., Rosendal T., Reid-Smith R., Friendship R. (2011). Longitudinal investigation of methicillin-resistant Staphylococcus aureus in piglets. Zoonoses Public Health.

[31] Tierschutzverordnung. https://www.fedlex.admin.ch/eli/cc/2008/416/de.

[32] Alt K., Fetsch A., Schroeter A., Guerra B., Hammerl J.A., Hertwig S., Senkov N., Geinets A., Mueller-Graf C., Braeunig J. (2011). Factors associated with the occurrence of MRSA CC398 in herds of fattening pigs in Germany. BMC Vet. Res..

[33] Broens E., Graat E., Van Der Wolf P., Van De Giessen A., De Jong M. (2011). Prevalence and risk factor analysis of livestock associated MRSA-positive pig herds in The Netherlands. Prev. Vet. Med..

[34] Broens E.M., Espinosa-Gongora C., Graat E.A.M., Vendrig N., van der Wolf P.J., Guardabassi L., Butaye P., Nielsen J.P., De Jong M.C.M., van de Giessen A.W. (2012). Longitudinal study on transmission of MRSA CC398 within pig herds. BMC Vet. Res..

[35] Sørensen A.I.V., Jensen V.F., Boklund A., Halasa T., Christensen H., Toft N. (2018). Risk factors for the occurrence of livestock-associated methicillin-resistant Staphylococcus aureus (LA-MRSA) in Danish pig herds. Prev. Vet. Med..

[36] Rantala M., Nurmi E. (1973). Prevention of the growth of Salmonella infantis in chicks by the flora of the alimentary tract of chickens. Br. Poult. Sci..

[37] Caly D.L., D’Inca R., Auclair E., Drider D. (2015). Alternatives to Antibiotics to Prevent Necrotic Enteritis in Broiler Chickens: A Microbiologist’s Perspective. Front. Microbiol..

[38] Agnolucci M., Palla M., Cristani C., Cavallo N., Giovannetti M., De Angelis M., Gobbetti M., Minervini F. (2019). Beneficial Plant Microorganisms Affect the Endophytic Bacterial Communities of Durum Wheat Roots as Detected by Different Molecular Approaches. Front. Microbiol..

[39] Allaart J.G., van Asten A.J.A.M., Vernooij J.C.M., Gröne A. (2011). Effect of Lactobacillus fermentum on beta2 toxin production by Clostridium perfringens. Appl. Environ. Microbiol..

[40] Dec M., Puchalski A., Urban-Chmiel R., Wernicki A. (2014). Screening of Lactobacillus strains of domestic goose origin against bacterial poultry pathogens for use as probiotics. Poult. Sci..

[41] Sikorska H., Smoragiewicz W. (2013). Role of probiotics in the prevention and treatment of meticillin-resistant Staphylococcus aureus infections. Int. J. Antimicrob. Agents.

[42] Mellmann A., Friedrich A.W., Rosenkötter N., Rothgänger J., Karch H., Reintjes R., Harmsen D. (2006). Automated DNA sequence-based early warning system for the detection of methicillin-resistant Staphylococcus aureus outbreaks. PLoS Med..

